# How home anterior self-collected nasal swab simplifies SARS-CoV-2 testing: new surveillance horizons in public health and beyond

**DOI:** 10.1186/s12985-021-01533-z

**Published:** 2021-03-20

**Authors:** Silvia Ricci, Lorenzo Lodi, Francesco Citera, Francesco Nieddu, Maria Moriondo, Valentina Guarnieri, Mattia Giovannini, Giuseppe Indolfi, Massimo Resti, Alberto Zanobini, Chiara Azzari

**Affiliations:** 1 Section of Pediatrics, Department of Health Sciences, University of Florence, Viale Gaetano Pieraccini 24, 50139 Florence, Italy; 2 Immunology and Molecular Microbiology Unit, Meyer Children’s Hospital, Viale Gaetano Pieraccini 24, 50139 Florence, Italy; 3 Section of Pediatrics, Department of Neurofarba, University of Florence, viale Gaetano Pieraccini 24, 50139 Florence, Italy; 4 Pediatric Department, Meyer Children’s Hospital, Viale Gaetano Pieraccini 24, 50139 Florence, Italy; 5 Meyer Children’s Hospital, viale Gaetano Pieraccini 24, 50139 Florence, Italy

**Keywords:** Self-swab, Nasal-swab, SARS-CoV-2 testing, Surveillance

## Abstract

The sample collection procedure for SARS-CoV-2 has a strong impact on diagnostic capability, contact tracing approach, ultimately affecting the infection containment performance. This study demonstrates that self-collected nasal-swab has shown to be a valid and well tolerated procedure to SARS-CoV-2 surveillance in a healthcare system. More significantly, no performance adequacy difference was detected in self-administered swabs between healthcare worker (HCW) and non-HCW which allows to speculate that this procedure could be successfully extended to the entire population for mass screening.

**Supplementary Information:**

The online version contains supplementary material available at 10.1186/s12985-021-01533-z.

## Introduction

A rapid, low cost and comprehensive SARS-CoV-2 testing strategy can provide enormous benefits to the containment effort of the current pandemic. Nasopharyngeal and/or oropharyngeal swab performed by a trained healthcare worker (HCW) is the gold standard procedure recommended by Centers for Disease Control and Prevention (CDC) [1]. This sampling approach has a high economic burden, it reduces the number of HCWs potentially available for other tasks, it fastens the depletion of personal protective equipment and exposes the HCWs to the risk of infection. Nonetheless, mid-turbinate or anterior nasal specimen’s collection procedures are recognized as valid [1–3], and there is growing evidence of the diagnostic reliability of self-collected swabs as a low-cost alternative to HCW-collected [4]. However, these data on the diagnostic accuracy of alternative procedures for SARS-CoV-2 testing are scarce. Recent studies were performed mostly on specific subjects’ groups as health care workers or on small sample sizes so that further assessment is needed before the broad implementation of these alternatives [4]. Notably, a potential extension to the general population is limited by the significant percentage of HCWs among participants of previous published studies and the low prevalence of positive tests [5, 6].

The present study aimed to assess the adequacy of unsupervised home self-collected nasal swabs using the expression of the human internal control gene *RNAse P* as an indicator of sampling quality performance. The study was developed in the context of the “UFFA!” project (UFFA! is the protocol submission code) for SARS-CoV-2 hospital active surveillance through simplified sampling procedures that may provide elements for the extension of self-collection nasal swab by non-HCWs.

## Methods

### Study design and participants

This work is a cross-sectional study, called UFFA!, started on 6th October 2020 and ended on 16th November 2020 at Meyer Children’s University Hospital (Florence, Italy).

The participants were no symptomatic HCWs (medical doctors and nurses) and non-HCWs (administrative personnel) working at Meyer Children’s University Hospital.

All participants joined the surveillance program on a voluntary basis.

Group A performed home self-collected nasal swabs. Control group (group B) received nasopharyngeal swabs performed by trained staff in the same period in the hospital dedicated swabbing center.

### Sample collection procedures

Group A (self-collection): HCWs and non-HCWs received a self-swab administration kit containing: a flocked tapered swab (ESwab, Copan, Brescia, Italy) and a tube, specimen labelling and transportation material (three-layer bag), and written instructions for the anterior nasal swab execution including a link to a video tutorial designed accordingly with international guidelines [1]. Self-swabbing had to be performed at home just before coming to work, possibly within 30 min, and the swab, contained in a three-layer packaging, had to be delivered in a dedicated box outside the laboratory of Immunology. This box was checked by laboratory staff, thus eliminating contact with potentially infected subjects. The analysis started within 30 min after the delivery.

Group B (controls): nasopharyngeal swabs were collected as recommended in international guidelines [1] from trained nurses at the hospital swabbing center. The group B participants were HCWs and non-HCWs.

### Laboratory analysis

The presence of human internal control gene *RNAse P* and SARS-CoV-2 RNA (N1 N2 and N3) in the samples was evaluated through quantitative reverse transcription-polymerase chain reaction (qRT-PCR), as described in CDC 2019-nCoV Real-Time RT-PCR Diagnostic Panel [7]. RNA was isolated and purified from 400 uL of nasal fluid specimens using MagCore Viral Nucleic Acid Extraction Kit (RBC Bioscience, Taiwan) according to manufacturer’s instructions. RNA is reverse transcribed to cDNA and subsequently amplified in the Applied Biosystems 7500 Fast Real-Time PCR Instrument using TaqPath™ 1-Step RT-qPCR Master Mix (Thermo Fisher scientific, USA) and N1, N2 and N3 primer and probe set [7]. Fluorescence intensity is monitored at each PCR cycle by Applied Biosystems 7500 Fast Real-Time PCR System with SDS version 1.4 software. The cycle threshold (CT) values of qRT-PCR are inversely related to the copy number of human or viral RNA.

The cycle threshold values of RT-PCR were used as indicators of the copy number of SARS-CoV-2 RNA. A cycle threshold value less than 40 is interpreted as positive for SARS-CoV-2 RNA and gene *RNase P*. If no increase in fluorescent signal is observed after 40 cycles, the sample is assumed to be negative.

### Satisfaction survey

We invited all group A participants to voluntarily answer a web satisfaction survey, including 4 items:

1. Procedure.
2. Home setting.
3. Time saved.
4. Instructions.

The intensity of the discomfort caused by the procedure was evaluated through a numeric pain scale ranging 1 to 10 both in the self-collected and staff-collected swab. Results were compared and the occurrence of adverse events was registered. The complete questionary is available as Supplementary material (Additional file 2).

### Statistical analysis

Data were processed with StatPlus:mac, AnalystSoft Inc. v7. Results were expressed as median and interquartile ranges (IQRs), as appropriate. The Mann–Whitney U test or Kolmogorov–Smirnov Test were used to compare group differences for continuous non-parametric independent samples. The categorical data were compared between groups using the χ^2^ test. p values < 0.05 were considered statistically significant.

## Results

Between October 6 and November 16, 2020, 827 adults (527 women, 77% F, mean age 40.7 ± 13.1) participated in the study, 578 were HCWs (70%) and 249 were no-HCWs (29%) (group A). Group B included 1437 (977 women, 68% F, mean age 46.2 ± 11.7).

Human internal control gene *RNAse P* was detected in 827/827 (100%) and in 1437/1437 (100%) subjects for group A and group B, respectively. No swabs were found to be invalid, considering the amplification of *RNase P* gene accordingly with CDC guidelines [7]. 

**Fig. 1:**
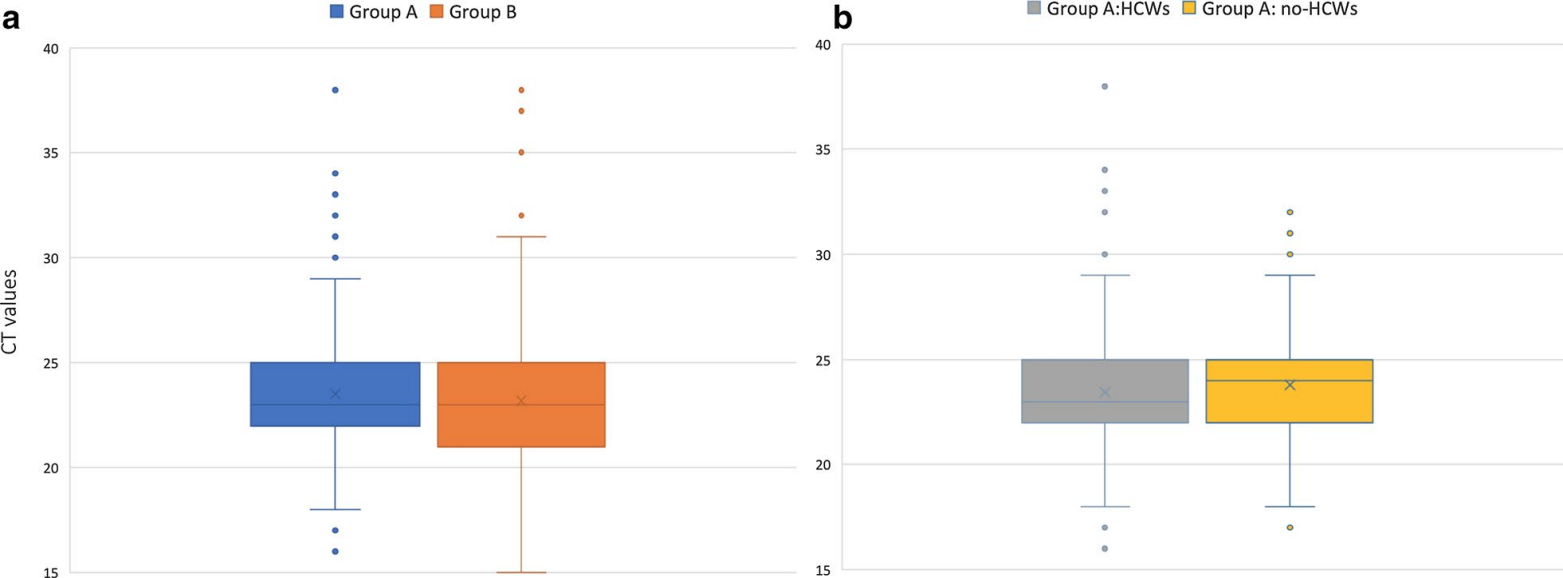
**a** The CT median values and IQRs of human internal control gene RNAse P in group A (blue), group B (orange) are not statistically different (Kolmogorov–Smirnov Test p 0.09). **b** The CT median values and IQRs of human internal control gene *RNAse P* group A HCWs (grey) and group A no-HCWs (yellow) are not statistically different (Kolmogorov–Smirnov Test p 0.6)

The median CT values for human internal control gene *RNAse P* were perfectly congruent in group A (self-collected swabs) and B (staff-collected swabs): respectively 23 (IQR 22.00–25.00) and 23 (IQR 21.00–25.00) (Fig. 1a).

Within group A, the expression of the gene *RNase P* showed similar median CT values in self-swabs performed by HCWs and non-HCWs with congruent IQRs: respectively 23 (IQR 22.00–25.00) and 24 (IQR 22.00–25.00) (Fig. 1b).

SARS-CoV-2 genome was detected in 11/827 self-collected swabs (positivity rate 1.33%) and 12/1437 staff-collected swabs (positivity rate 0.8%) with no statistically significant difference (X^2^ p 0.27, OR 95% 1.58 CI 0.6928—3.59). The CT median values for N3 SARS-CoV-2 were 18.5 (IQR 15.5 -25.25) in group A and 21 (IQR 16.5–28.00) in group B (Mann–Whitney U test p = 0.58) (Additional file 1: Fig. S1). All positive resulted self-collected swabs were subsequently (after 24–48 h) confirmed positive by staff-collected swab, but no quantitative RT-PCR has been done for the confirmation molecular test.

### Survey results

The tested subjects who participated in the survey were 490/827 (59%). Among participants, 92.5% were highly satisfied with self-collection swabbing at home (overall satisfaction score mean value 4.62 ± 0.69 SD), 99.2% of the participants stated that the procedure was easy to perform and 95.8% found the instructions very clear. One of the most appreciated aspects was the time saved, with 96.5% of participants who declared to have saved time compared to arranging an appointment for the staff-collected swab at the hospital and 95.1% were extremely satisfied of this aspect. The discomfort perceived during nasal self-swabbing was significantly lower than that perceived during staff-collected nasopharyngeal swabbing (mean values ± SD, 2.7 ± 1.6 vs 6.22 ± 1.16; Kolmogorov–Smirnov Test p < 0.0001). Two participants reported mild, self-limiting epistaxis after the procedure. No other adverse events were reported by participants after nasal swab self-collection at home.

## Discussion

We demonstrated that nasal self-collected specimens were highly comparable to staff-collected nasopharyngeal specimens in terms of collection adequacy. SARS-CoV-2 genome detection rate between two groups A and B was equivalent. Considering the low prevalence of SARS-CoV-2 infection among the hospital personnel, the study would not have obtained enough positive results to validate the procedure in terms of sensitivity and specificity. This limitation, frequently encountered in literature, was overcome by using the human internal control gene *NAse P* as an indicator of adequate swabbing performance. All specimens had detectable *RNase P*, CT values for *RNase P* and SARS-CoV2 RNA detection were almost identical in self-collected swabs compared with CT observed in staff-collected nasopharyngeal swabs. The magnitude of the CT differences, when present (Group A HCWs *vs* no-HCWs), was minimal (ΔCT = 1) and comparable to the difference between CT values that can be found if the same sample is analyzed twice at the same conditions. 

The diffusion of anterior nasal swab home self-administration is undoubtedly time saving and allows a minor deployment of HCWs which is crucial during healthcare emergencies. This approach would reduce costs in terms of staff employed and PPE used, allowing at the same time an easier access to the test and thus enhancing contact tracing and reducing the risk of infection for patients and HCWs. Another advantage of the unsupervised home self-administration is the possibility to longitudinally follow the infectiveness of the infected home-isolated patients, saving on specific PPE, avoiding the access to the infected person's residence and therefore dramatically decreasing the risk of exposure. Previous studies have described a good accuracy of self-swabbing for influenza detection [8, 9] and for SARS-CoV-2 [5, 6]. These results demonstrate that there is no difference between HCWs or non-HCWs in the accuracy of unsupervised home self-collected nasal swab, solving one of the major limitations [4, 5] of available data and opening-up to the possibility of self-administration to the general population.

We are aware of the limitations of this analysis. The study design is limited by the impossibility to determine the sensitivity of the method: the subjects performed self-collected nasal swab did not received swab administered by HCWs at the same time (gold standard procedure). The conceived study design is forcibly derived from the emergency conditions of the pandemic and its high impact on healthcare system: duplicate testing was not feasible in terms of PPE use, collection materials, reagents and laboratory commitment for screening procedure.

However, the work aims to prove that this procedure may play a pivotal role in simplifying and empowering active surveillance in hospital setting where extensive testing is critical to prevent SARS-CoV-2 transmission for no symptomatic HCWs and no-HCWs. Self-swabbing procedure could help screening a large number of subjects simultaneously, allowing prevalence point studies for hospital and other work settings. The savings in terms of HCWs commitment allows this type of screening to be performed much more frequently than the canonical method.

It can be possible to speculate that this procedure could be successfully extended to the general population for mass screening of no suspected COVID cases. Ongoing studies at Meyer Children’s Hospital are evaluating the possibility to validate self-swabbing procedure with more rapid antigenic detection methods and whether it will be possible to apply this procedure in the pediatric population, for example as a periodic screening in the schools.

## Conclusions

This study demonstrates that self-collected nasal-swab has shown to be a feasible and well tolerated procedure to SARS-CoV-2 screening program in a healthcare system. More significantly, no performance adequacy difference was detected in self-administered swabs between HCW and non-HCW which allows to speculate that this procedure could be successfully extended to the entire population for mass screening.

## Supplementary Information

**Additional file 1: Figure S1**. The CT median values and IQRs of SARS-CoV-2 RNA in group A (blue), group B (orange): are not statistically different (Mann–Whitney U test p = 0.58).

**Additional file 2.** Complete satisfaction survey.

## Data Availability

Data and materials are available on request.

## Abbreviations

SARS-CoV-2: Severe acute respiratory syndrome coronavirus 2
HCW: Healthcare worker
CDC: Centers for Disease Control and Prevention
qRT-PCR: Quantitative reverse transcription-polymerase chain reaction
CT: Cycle threshold
IQRs: Interquartile ranges
