# Supplementary material for: How home anterior self-collected nasal swab simplifies SARS-CoV-2 testing: new surveillance horizons in public health and beyond
Source: Virol J. 2021 Mar 20;18:59. doi: 10.1186/s12985-021-01533-z (PMC7980800; doi:10.1186/s12985-021-01533-z)
Supplement: Supplementary file 1 — Additional file 1: Figure S1. The CT median values and IQRs of SARS-CoV-2 RNA in group A (blue), group B (orange): are not statistically different (Mann–Whitney U test p = 0.58). [file 12985_2021_1533_MOESM1_ESM.pptx]

## Slide 1
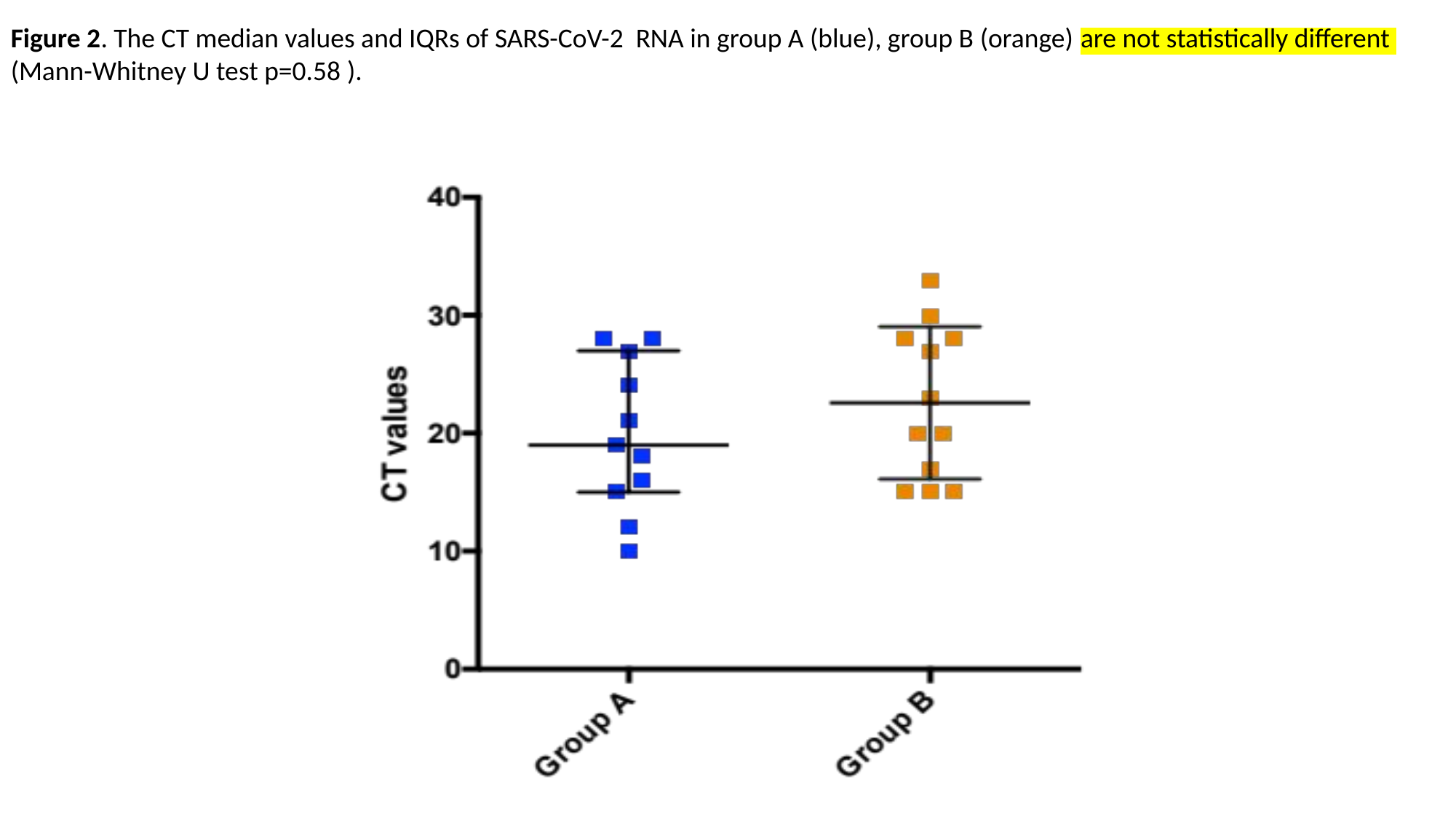

Figure 2. The CT median values and IQRs of SARS-CoV-2 RNA in group A (blue), group B (orange) are not statistically different
(Mann-Whitney U test p=0.58 ).
