# Supplementary material for: How home anterior self-collected nasal swab simplifies SARS-CoV-2 testing: new surveillance horizons in public health and beyond
Source: Virol J. 2021 Mar 20;18:59. doi: 10.1186/s12985-021-01533-z (PMC7980800; doi:10.1186/s12985-021-01533-z)
Supplement: Supplementary file 2 — Additional file 2. Complete satisfaction survey. [file 12985_2021_1533_MOESM2_ESM.docx]

UFFA! Satisfaction survey

1. How satisfied are you with performing the procedure at home compared to performing it at the health center?

1 (Very Dissatisfied) 2 3 4 5 (Very Satisfied)

1. How satisfied are you for paper instructions provided and the video tutorial?

1 (Very Dissatisfied) 2 3 4 5 (Very Satisfied)

1. Do you think this procedure turned out to be easy?

Yes No

1. Do you think you have saved time compared to performing a pharyngeal swab with a scheduled appointment at a swab center (either at the hospital through health surveillance or in the territory)?

Yes No

1. If you think you have saved time, how satisfied are you with this aspect?

1 (Very Dissatisfied) 2 3 4 5 (Very Satisfied)

1. How much pain did you experience from 1 to 10 for self-collected nasal swab?

1 2 3 4 5 6 7 8 9 10

1. How much pain did you experience from 1 to 10 for nasopharyngeal swab performed by health care provider?

1 2 3 4 5 6 7 8 9 10

1. Did any adverse events occur during the self-collection swab?

Yes No

1. If so, which ones?

……………………………………………………………………………………………………………………………………….

1. How satisfied are you overall with this method of swab collection (self-collected nasal swab)

1 (Very Dissatisfied) 2 3 4 5 (Very Satisfied)

1. Want to leave us with some suggestions for improvement?

……………………………………………………………………………………………………………………………………….
